# Annual Address

**Published:** 1878-11

**Authors:** L. G. Noel


### ANNUAL ADDRESS.

*Delivered before the Tennessee Dental Association, June 26, '78.*

BY DR. L. G. NOEL.

GENTLEMEN OF THE TENNESSEE DENTAL ASSOCIATION:—It is my pleasant duty to address you upon this occasion as your presiding officer and being one of the youngest members of the society, I feel a double sense of the responsibility of the task which I am about to essay.

The annual address of the president of a society like ours, should, if I understand it, be a kind of executive message, in which the work of the body is resumed and new work mapped out. The scope and compass of my scheme, therefore, compels me to be comprehensive, general and brief.

On the 24th of June, 1867, a meeting of a few of the leading dentists of Memphis, was held in that city for the purpose of taking steps to call a convention of the profession of the State with a view to the organization of a permanent society. That convention was accordingly assembled in the city of Nashville on the 26th of July following, and thus our association began. In retrospecting the period of eleven years that have elapsed since that primary assemblage and comparing our progress with that so slowly and laboriously made before the existence of our society, we can not repress a feeling of pride in our record; and of gratitude to its founders, but at the same time we are painfully conscious of shortcoming in our work.

That achieved by similar organizations in other states, so far transcends it as to completely humiliate us in the comparison.

The number of students sent from our state to the dental colleges of the country, has perceptibly increased from year to year, and this we believe largely due to the influence of this organization upon the dentists of Tennesee, but we know we ought to-day to have no young man in the state, contemplating an entrance into the profession by any other than "the door of the sheep fold"

Do they ever climb up any other way? I have only to point you to the cards of self styled doctors in every country newspaper in the state. If the profession in Tennessee had been fully organized and had worked with singleness of purpose, laws would long ere this have been enacted by our legislature so regulating the practice of dentistry in the state as to brand every self constituted dentist in the state with the mark of the thief that climbed up by some other way. I would urge upon the society the importance of this matter of protective legislation, and suggest the appointment of a committee to present it to the general assembly of the state at its next meeting.

I know that some effort was made to secure the passage of protective laws some years ago, but we should not be discouraged and give over because of one or two failures. Another matter that I would call your attention to, is the existence of two separate and distinct dental societies in the state where there ought to be but one, and the unfortunate localization of those societies in the Eastern and Middle divisions and West Tennesee, from which sprang much of the youthful vigor of our organization, and to which legitimately belongs whatever of ripened fruit it may bear, has almost entirely lost interest in the work, but would we think, be aroused to some degree of action, if we could secure a fusion in the East Tennessee Association with our own, making it truly a state society such as it ought to be. A correspondence with some of the leading members of the Eastern Tennessee Association convinces us of their readiness to co-operate with us to this end.

I therefore would suggest that you invite the East Tennesee Society to hold a joint meeting with us next year with a view to a fusion of the two organizations, if it shall then appear expedient and agreeable. Let us continue to call upon West Tennesee to help us, for if once more enlisted, she can give as valuable assistance. We can not go into the work before us, bereft of so valuable a member. The eye can not say to the hand, I have no need of thee. This matter of consolidation of all such organizations in the state, is most fit and opportune just now in view of the effort about to be made at Niagara, in August, to secure a coalition of the several dental societies in the United States, into one National Dental Congress, to which, states are to send their representatives.

Being in favor of this national union of societies, and of the sectional work as proposed by its originators, I would urge upon you the selection of good and efficient delegates, who will go to Niagara and work vigorously to this end.

Now in conclusion, there is one other question I would bring before you as worthy of discussion, indeed, it thrusts itself before us as almost demanding consideration by this body—viz: the establishing a Dental Department in Vanderbilt University. Speaking for the University, we think we may say she is ready to take the infant Department to her maternal bosom as soon as you may organize and equip it.

The question for us to consider is the feasibility and probable success of such an attempt at this time. Three of the great universities of America have introduced this department, thereby recognizing dentistry as one of the learned professions. Those who have watched the progress of education in this country, have noted the increasing strength of the university system pari passue with the decadence of the collegiate. All will, we predict, be in a few years collateral to the university system; hence future success for a young institution would seem to depend upon such an alliance. I would anticipate the objection often urged, that there are already too many dental colleges, by calling your attention to the fact, that most of these are located in North Eastern cities; they do not supply the wants of the West and South. It is not my purpose to argue the case before you, but merely to present the question for your consideration. Perhaps it is not a question that this Association can handle at this time in any practical way, but no harm can be done by discussing that, which is agreed by all to be a desired consummation.

